# Quantum dots: a new tool for anti-malarial drug assays

**DOI:** 10.1186/1475-2875-10-118

**Published:** 2011-05-09

**Authors:** Min-Je Ku, Fernando M Dossin, Youngseon Choi, Carolina B Moraes, Jiyoung Ryu, Rita Song, Lucio H Freitas-Junior

**Affiliations:** 1 Center for Neglected Diseases Drug Discovery (CND3), Institut Pasteur Korea, Seongnam-si, Gyeonggi-do, South Korea; 2 Nano/Biochemistry Group, Institut Pasteur Korea, Seongnam-si, Gyeonggi-do, South Korea

## Abstract

**Background:**

Malaria infects over 300 million people every year and one of the major obstacles for the eradication of the disease is parasite's resistance to current chemotherapy, thus new drugs are urgently needed. Quantum dot (QD) is a fluorescent nanocrystal that has been in the spotlight as a robust tool for visualization of live cell processes in real time. Here, a simple and efficient method using QD to directly label *Plasmodium falciparum*-infected erythrocytes (iRBCs) was searched in order to use the QD as a probe in an anti-malarial drug-screening assay.

**Methods:**

A range of QDs with different chemical coatings were tested for their ability to specifically bind iRBCs by immunofluorescence assay (IFA). One QD was selected and used to detect parasite growth and drug sensitivity by flow cytometry.

**Results:**

PEGylated-cationic QD (PCQD) was found to specifically label infected erythrocytes preferentially with late stage parasites. The detection of QD-labelled infected erythrocytes by flow cytometry was sensitive enough to monitor chloroquine anti-malarial toxicity with a drug incubation period as short as 24 h (EC_50 _= 113nM). A comparison of our assay with another widely used anti-malarial drug screening assay, the pLDH assay, showed that PCQD-based assay had 50% improved sensitivity in detecting drug efficacy within a parasite life cycle. An excellent Z-factor of 0.8 shows that the QD assay is suitable for high-throughput screening.

**Conclusions:**

This new assay can offer a rapid and robust platform to screen novel classes of anti-malarial drugs.

## Background

Malaria remains a major global health problem, threatening over 300 million people and causing nearly one million deaths annually [1]. It is still a major cause of death especially in children under five years of age and pregnant women in areas such as sub-Saharan Africa, which bear the greatest burden of the disease. Due to the emergence of resistance, the rate in which the available drugs fail in the treatment of malaria has increased. To worsen this situation, recent reports on the emergence of resistant strains to artemisinin-based combination therapy (ACT) [2,3] have accelerated the urgent need of new drugs.

Recently, the CdSe/ZnS semiconductor nanocrystal known as quantum dot (QD) has been widely used for various bioimaging applications as well as *in vitro *diagnostics due to high photostability, large stokes shift, and tunable narrow emission spectral characteristics [4]. These particular interesting fluorescent properties of QDs allowed it to be used as a robust fluorophore for labelling bacteria [5], red blood cells (RBCs) [6], various intracellular organelles [7], genes [8,9], and proteins [10]. In particular, Tokumasu *et al *[11] used antibody-conjugated QDs to show the distinct pattern of distribution of protein and to observe erythrocyte membrane deformation occurring during the invasion of erythrocytes by *P. falciparum*. To search for a QD that could specifically label *P. falciparum*-infected RBC, a range of QDs with different chemical coatings (-COOH, -NH_2_, amino PEG, methoxy PEGs) were tested. A PEGylated cationic QDs (PCQD), which have been successfully implemented for cellular labelling [12], was found to specifically label the iRBC, particularly at late stage parasites. Herein, the present study shows (i) a simple and efficient method to label *P. falciparum*-infected RBC using a QD-based probe and (ii) its applicability as an efficient probe for anti-malarial drug screening.

## Methods

### QD synthesis

The PCQD were prepared by ligand exchange of trioctylphospine oxide (TOPO)-QD with DEDEA (dihydrolipoic acid-2,2'-ethylenedioxy bis(ethyleneamine)) and subsequent conjugation with N-hydroxyl succinimide methoxy PEG2000 (NHS-mPEG2000) molecules as reported previously [12]. In addition to the positively charged PCQD, negatively charged QDs with chemical coatings of carboxylated polymers (QD-COOH), amino PEG2000 (QD-PEG-NH_2_), methoxy PEG2000 (QD-mPEG) were also prepared using polymer-coating method with some modifications [13,14]. See Additional file 1 for the detailed synthetic procedures and characterization methods of QDs.

### *Plasmodium falciparum *culture

*Plasmodium falciparum *3D7 strain was maintained *in vitro *in human O^+ ^erythrocytes at 3% haematocrit in RPMI 1640 media supplemented with L-glutamine, 25 mM HEPES and sodium bicarbonate. Additionally, 0.5% Albumax I, 0.1 mM hypoxanthine and 16 μM thymidine were added to complete the media. To obtain a synchronized culture, the parasites were submitted to two sequential 5% sorbitol treatments that were 10 hours apart, cultivated for an additional 30 hours, allowed to re-invade RBCs and then used for experiments at 10-15 hours post-invasion (hpi) for young-stage parasites and at 30-35 hpi for late-stage parasites.

### Fluorescence image acquisition

Synchronized young and late stage parasites were incubated with PCQD and Hoechst (RBC: PCQD: Hoechst = 5 × 10^7 ^cells: 2 μM: 5 μM) for 1 h at 37°C with gentle shaking. Parasites were then washed once with PBS and transferred to glass slides (Cell-line/Erie Scientific Co, US) for image acquisition. Image acquisition was performed on Epifluorescence microscope (Eclipse 90i, Nikon, Japan) using synchronized parasites.

### Flow cytometry

To determine the sensitivity of PCQD in detecting different levels of parasitemia, synchronized late-stage parasites were serially diluted with fresh RBC and analysed in a Flow cytometer FACSCanto II (BD, USA). For the procedure, cells were fixed with 2% paraformaldehyde + 0.008% glutaraldehyde solution for 30 min, washed once in PBS and stained with PCQD and Hoechst as described for the image acquisition experiments. The cells in the gate of both PCQD and Hoechst positive were only counted for data analysis. As a control, slides using the same serially diluted samples were prepared, stained with Giemsa (Merck) and counted manually using a light microscope.

### Dose-response curve of chloroquine

Dose-response experiments (range 0.45 nM to 1 μM) were carried out in 96-well plate at 0.5% and 10% parasitaemia (for 60 h and 24 h drug-exposure, respectively) of synchronized parasites (young stage) at 2% haematocrit, 37°C, in triplicates. After the drug exposure, the cells were fixed and stained as already described. Data analysis was performed using FACSDiva Software (BD, USA). The data were normalized using average result of chloroquine-treated wells (at EC_100 _concentration) and average result of non-treated wells as 0% and 100%, respectively.

For the pLDH assay, the same conditions were used but culture was added to 384-well plate (Greiner). After the drug exposure time was finished, the plates were frozen overnight at -20°C. After thawing, the plates were shaken for 45 sec at 1700 rpm in Mix Mate (Eppendorf) and 5 μL of the lysate was transferred into the correspondent well of another plate containing 30 μL of Malstat Reagent [15] and incubated for 2 h. The absorbance (650 nm) was read at Spectramax M5 (Molecular Devices, USA).

## Results

### Specificity and sensitivity of PCQD for detecting *P. falciparum*-infected erythrocytes

**Figure 1:**
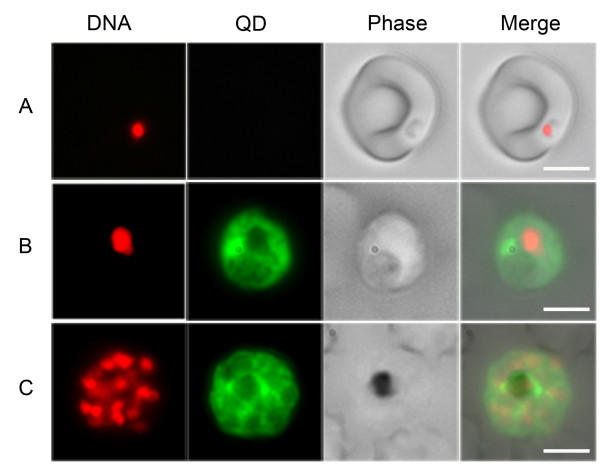
**Quantum dot (QD) labelling on *P. falciparum-*infected erythrocytes showing that only late-stage iRBCs are labelled**. Early-stage (ring) iRBCs (A) are not labelled by the QD, while the late-stage trophozoite (B) and segmented schizont (C) iRBCs are both labelled. The parasites were stained with Hoechst 33324 (in red, first column from the left) and PCQD (in green, second column). Phase contrast images (third column) and merged images (fourth column) are also shown. Bars, 5 mm.

A panel of QDs with different chemical coatings (QD-COOH, QD-PEG-NH_2_, QD-mPEG, and PCQD) were characterized with regard to their hydrodynamic sizes, charges, and quantum yield (Table 1). These well-defined QDs with different charges and sizes were visually screened for their ability to bind iRBCs, and PCQD was found to bind *P. falciparum*-infected erythrocytes (Figure 1). However, this PCQD was only able to bind mature asexual stage-iRBCs, i.e., the pigment-bearing trophozoites and schizonts, and not young stage-iRBCs. The quality of the labelling (signal to noise ratio) was enough for clear identification of positives and quantitatively, 60% of the infected erythrocytes were PCQD-positive.

**Table 1:** Molecular characteristics of QDs used in this study

Sample code	**Hydrodynamic size (nm)**^**a)**^	**Zeta potential (mV)**^**b)**^	**Quantum Yield**^**c)**^
PCQD	10 ± 0.8	20 ± 3	0.1
QD-COOH	10 ± 1.2	- 33 ± 6	0.2
QD-PEG-NH_2_	13 ± 1.8	-25 ± 5	0.2
QD-mPEG	12 ± 1.3	- 8 ± 1	0.2

^a) ^Number-averaged diameter measured using dynamic light scattering instrument (n = 3), ^b) ^average of three measurements in water, and ^c) ^compared with fluorescein (quantum yield = 0.95).

**Figure 2:**
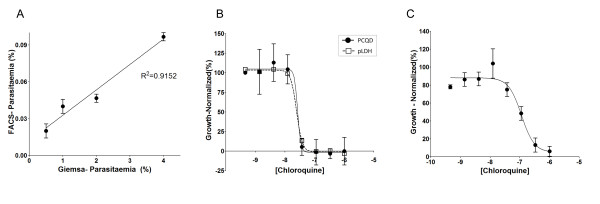
**Validation of PCQD as a tool for *P. falciparum *drug discovery**. (A) Comparison of QD detection of increasing *P. falciparum *parasitaemia assessed by flow cytometry and manually from Giemsa-stained culture smears. The late-stage *P. falciparum *culture was serially diluted and incubated with PCQD and Hoechst and analyzed by flow cytometry. The plot shows the correlation (R^2 ^= 0.9152) of the parasitaemia values detected by both methods. (B) Comparison of chloroquine DRCs using PCQD and pLDH assays. Synchronized young parasites were incubated with increasing concentrations of chloroquine. After 60 h of incubation, samples were either fixed and labelled with PCQD and Hoechst for PCQD assay or developed for the pLDH assay as described above. The plot shows a consistent EC_50 _of 26 nM for both assays. (C) The chloroquine DRC of a 24-h drug exposure within a single erythrocytic cycle using the PCQD assay. The DRC-derived EC_50 _was 113 nM.

To verify its labelling sensitivity, synchronized late-stage parasites were serially diluted to achieve a linear curve of parasitaemia and incubated with PCQD and the DNA staining Hoechst for labelling. The number of PCQD-labelled cells was measured by flow cytometry and, indeed, decreased proportionally as the number of the parasites was diminished, confirming that PCQD was specifically binding to iRBCs. In agreement with these findings, a high linear correlation (R^2 ^= 0.9152) was observed between cytometry data and manual assessment of parasitaemia from Giemsa-stained smears for each dilution point (Figure 2A).

### PCQD as a probe in chloroquine dose-response assay for 60 h drug exposure

The specific binding of PCQD to infected RBCs prompted us to setup an assay for anti-malarial drug screening using PCQD as a fluorescent probe. Early stages synchronized *P. falciparum *cultures at low parasitaemia were incubated in triplicates with serially diluted concentrations of chloroquine to obtain a dose-response curve (DRC). To compare PCQD assay with the pLDH assay, a well-known anti-malarial drug-screening assay, the same cultures were prepared in 384-well plate in parallel. After 60 hours of drug exposure, the cultures from each plate were developed by PCQD assay and pLDH assay as described in Methods. The DRC of chloroquine from both assays showed EC_50_s of 26 nM, in accordance with reported values using other assays (Figure 2B) [16]. In addition, the Z-factor calculated for the PCQD assay was 0.8, which is high enough to enable assay automation and high-throughput screening.

### PCQD as a probe in chloroquine dose-response assay for 24 h drug exposure, within a single asexual cycle

Chloroquine exerts its plasmocidal effects only on mature stages of the asexual cycle [16]. Thus we further tested if PCQD would be sensitive enough to detect chloroquine effects on parasite viability within a single asexual cycle (i.e., without changes in the parasitaemia). Synchronized early stage parasites at high parasitaemia (10%) were incubated for 24 h with serial dilutions of chloroquine followed by flow cytometry analysis. The DRC-derived EC_50 _was 113 nM, which was also comparable with previous reports (Figure 2C) [16]. A paralleled pLDH assay was also performed and compared with PCQD assay, showing PCQD-based assay was significantly more sensitive in detecting drug efficacy within a parasite life cycle than in the pLDH assay; at 100 nM chloroquine, PCQD assay detected 53%(±19) while pLDH assay showed 0%(±8) of growth inhibition (p = 0.0024, two tailed t-test).

## Discussion

QDs have been largely applied in conjugation with various biomolecules including DNA oligonucletides, peptides, and antibodies due to their properties such as photostability and narrow emission spectra [17,18]. In this work, a range of different QDs were screened for labelling the iRBC to use it as a probe in an assay to search for anti-malarial drugs. This straightforward strategy of searching for a QD that could by itself specifically label *P. falciparum *iRBC shortened the assay development time, sparing us, for example, from the development and characterization of an antibody against parasite-specific protein and antibody-QD conjugation processes.

The QDs used for this study include various surface coatings with different charges (positive or negative), PEG molecules (with or without), and functional groups (carboxyl or amine). Out of the various QDs tested, only PCQD with a positive and PEGylated coating could interact specifically with late stage parasitized RBCs. Although the nature of the interaction between the PCQD and the iRBC is yet not clear, the fact that negatively charged QD did not label RBC infected with late stage parasites suggests that the positive charge might be important for this interaction. In this sense, positively charged QD has been known to facilitate the binding with cellular membrane via electrostatic interaction. The PCQD originally developed in our lab has strong positive charge in the surface with high positive zeta potential value and this positive charge seemed to promote the interaction with the cell membrane [10,12]. In addition, given (i) the surface pattern of the labelling observed, (ii) the size of the PCQD that does not allow for free diffusion across the membrane and (iii) the absence of endocytosis in red blood cells it is likely that the interaction between PCQD and the iRBC takes place only at the cell's surface although the target(s) for this interaction remains unknown.

The specific labelling of infected RBCs by PCQD prompted us to test if the PCQD could be used as a probe to determine drug efficacy. The PCQD-based assay was able to detect successfully the effect of chloroquine not only in 60 h but also in 24 h, within a parasite life cycle, with 50% more sensitivity than pLDH assay. The PCQD assay can provide an alternative, rapid and efficient method for screening fast-acting anti-malarial drugs because of its ability to detect the plasmocidal effects of a drug within a single erythrocytic cycle. Its cost, comparable to that of pLDH assay, makes it accessible to practically all academic set-ups and its performance (z' = 0.8) enable for high-throughput setting as well. Furthermore, due to the PCQD's exclusive labelling of late-stage parasites, this assay may be a powerful tool to look for specific drugs interfering in the maturation of parasites. This possibility is currently under investigation.

## Conclusions

Although the mechanism of specific labelling of parasitized RBC at the late stage with the PCQD has to be further elucidated, the ability of the PCQD to label the live parasitized RBC with high photostability led us to develop a rapid and efficient assay to screen anti-plasmodial compounds, setting a platform to search novel anti-malarial drugs.

## Competing interests

The authors declare that they have no competing interests.

## Authors' contributions

MK carried out FACS analysis, performed the chloroquine dose-response assay and drafted the manuscript. FMD performed pLDH assay, designed and participated in chloroquine dose-response assay. CBM carried out IFA and participated in chloroquine dose-response assay. JR and YC prepared PCQD including QDs with different chemical coatings. MK, FMD and CBM completed the final version of the manuscript. RS and LH approved the final manuscript. All authors read and approved the final manuscript.

## Supplementary Material

Additional file 1 **QD synthesis and characterization**. Detailed explanation of QD synthesis and methods used for their characterization as seen in Table 1. Click here for file
