# Supplementary material for: Quantum dots: a new tool for anti-malarial drug assays
Source: Malar J. 2011 May 9;10:118. doi: 10.1186/1475-2875-10-118 (PMC3112454; doi:10.1186/1475-2875-10-118)
Supplement: Additional file 1 — QD synthesis and characterization. Detailed explanation of QD synthesis and methods used for their characterization as seen in Table 1. [file 1475-2875-10-118-S1.DOC]

**Additional file 1**

**Synthesis and characterization of QDs used in this study**

**Materials**

Cadmium nitrate (99.99%), sodium myristate (99%), octadecyl phosphonic acid (ODPA) hexamethyldisilathiane ((TMS)2S), diethylzinc (ZnEt2), trioctylphosphine (TOP, 90%), trioctylphosphine oxide (TOPO, 99%), selenium powder (99.9%), 2,2’- (ethylenedioxy)bis(ethylamine), and trifluoroacetate (TFA) were purchased from Aldrich. NHS-methoxy PEG2000 was purchased from SunBio (Korea). All the reagents were used as received.

**Characterization methods**

All absorption spectra were obtained in a quartz cuvette using Cary 5000 UV-Vis spectrometer (Varian Inc., CA, USA) with a spectral range of 200 – 800 nm, a scan rate of 600 nm/min and a slit width of 2 nm. Photoluminescence was measured by Fluorolog®-3 spectrofluorimeter (HORIBA, Jobin Yvon, UK) with excitation at 405 nm at an integration time of 0.2 s and an excitation and emission slit width of 2 nm. Dynamic light scattering (DLS) and zeta potential measurements were performed in DI water at room temperature using Nano-ZS (Malvern, UK).

**Synthesis of TOPO-capped CdSe/ZnS QDs (QD-TOPO)**

The QD-TOPO was prepared by the method reported in the literature with a slight modification^1^. Briefly, a mixture of CdO (0.20 mmol), ODPA (0.205 mmol), and technological grade TOA (10 ml) was heated to 300°C under Ar purging to get a clear solution. A 2-g solution of Selenium powder (0.2 mmol, dissolved in 2 ml of TOP) was quickly injected into this hot solution, and then the reaction mixture was allowed to cool to 250°C for the growth of CdSe nanocrystals. Serial quantitative aliquots were taken out at different reaction times for kinetic studies to monitor the progress of the reaction by measuring the UV-vis absorbance. The reaction was stopped when when the reaction has completed to aquire QDs with desired emission wavelength. For a typical synthesis of CdSe/ZnS core shell QD, 2mL of the crude solution of CdSe QDs were mixed with 3 g of TOPO and 3 g of HDA, and heated to 160°C. The Zn and S precursor solution was then added dropwise to the vigorously stirred solution of CdSe QDs for 3 h after which the solution was cooled down to room temperature. The solution was added 40 mL methanol to each tube and centrifuged for 30 min at 4,000 g at room temperature. After dissolving TOPO- CdSe/ZnS in chloroform, the solution was filtered through a 0.2 μm pore size polypropylene syringe filter.

**Synthesis and characterization of PEGylated QD-DPA**

The starting CdSe/ZnS QD was water-solubilized through ligand exchange reaction using a carefully designed DHLA-derived ligand, DPA (DHLA-2,2’-(ethylenedioxy) bis(ethylamine)) as we previously reported ^2, 3^. To enhance water-solubility and the stability in various biological buffers such as PBS, the terminal amino group of the QD-DPA was further conjugated with NHS-mPEG2000 molecules (Scheme 1)


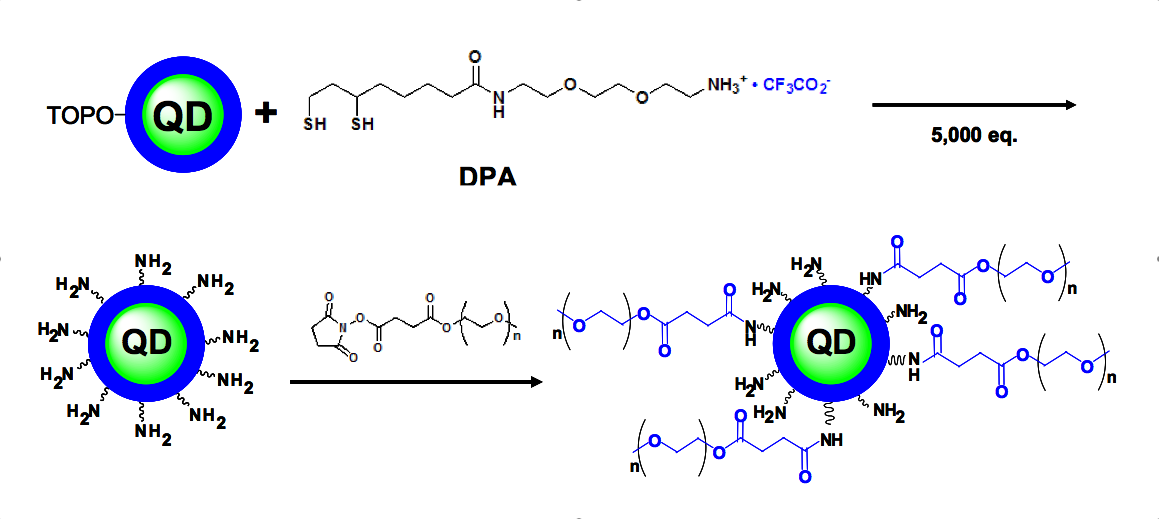


Scheme 1. Synthesis of PEGylated QD-DPA (QD-DPA-PEG2k)

**Synthesis and characterization of the polymer-coated QDs (pcQD-COOH)**

In addition to the positively charged amine-functionalized QD-DPA, negatively charged QD were also prepared using polymer-coating method reported in the literature with a slight modification^4, 5^. Briefly, trioctylphosphine oxide (TOPO)-capped CdSe/ZnS QDs were encapsulated with amphiphilic polymer molecules (octylamine-moeified polyacrylic acid) via hydrophobic interactions.

*Synthesis of octylamine-modified poly (acrylic acid)*. Poly (acrylic acid) (PAA, 3.6 g; M.W. 1800, Aldrich) and EDC (2.3 g) were transferred into a 100 mL round-bottom flask. 40mL of DMF was added to dissolve the mixture. About 1.2 mL of octylamine (99 %, Aldrich) was added dropwise into the reaction flask. When the reaction was complete, DMF was removed by evaporator, and the residue was mixed with 10mL of acetone and transferred into a centrifuge tube. 40 mL of water was added, and the gummy precipitated product was separated by centrifugation (3000 rpm for 10 min) and washed thoroughly with water. The amount of octylamine conjugation was ~ 15 % as determined by NMR analysis. The octylamine-modified PAA with this range of ratio showed the best water solubility and the highest quantum yield (QY) of 30 % ~ 60 %.

*Polymer coating of TOPO-capped CdSe/ZnS.* The octylamine-modified PAA was dissolved in 2 mL of chloroform. Tetramethyl-ammonium hydroxide was added to the polymer solution to raise the pH of the solution up to 10. The polymer solution was added to 5 mL of chloroform in a 20 mL round bottom flask and the solution was stirred for 1 min to ensure complete admixture of the polymer solution. With continued stirring, CdSe/ZnS QD solution was added dropwise to the polymer solution. The mixed solution was then stirred for two minutes to ensure the complete homogeneous solution and then the chloroform was removed in vacuo with a slight heating to yield a thin film of the particle-polymer complex on the wall of the flask. 5 mL of distilled water

was added to the flask and swirled along the walls of the flask to disperse the particles in the aqueous medium. The dispersion was then allowed to stir overnight at room temperature. Finally, the solution was centrifuged (4,000 g, 15 min) to remove aggregates and filtered through a 0.2 μm pore size PVDF syringe filter. To purify the polymer-coated QDs from remaining polymer, gel permeation chromatography was performed using the UltrahydrogelTM 1000 and 2000 columns at the flow rate of 1ml/min at 35^o^C in 50 mM borate buffer (pH 8.0). The chromatogram was recorded at 254 nm using Waters 2482 UV-detector. The resulting polymer-coated QDs in water were further used for PEG conjugation.


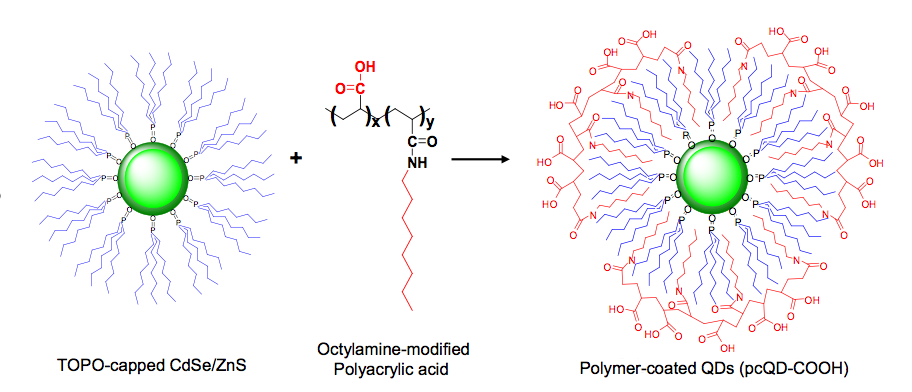


Scheme 2. Synthesis of polymer-coated QDs (pcQD-COOH)

**References**

1. Jun, S.; Jang, E., Interfused semiconductor nanocrystals: brilliant blue photoluminescence and electroluminescence. Chemical Communications 2005, 4616-4618.

2. Lee, J.; Kim, J.; Park, E.; Jo, S.; Song, R., PEG-ylated cationic CdSe/ZnS QDs as an efficient intracellular labeling agent. Physical Chemistry Chemical Physics 2008, 10, (13), 1739- 1742.

3. Lee, J.; Choi, Y.; Kim, J.; Park, E.; Song, R., Positively Charged Compact Quantum Dot- DNA Complexes for Detection of Nucleic Acids. Chemphyschem 2009, 10, (5), 806-811.

4. Gao, X. H.; Cui, Y. Y.; Levenson, R. M.; Chung, L. W. K.; Nie, S. M., In vivo cancer targeting and imaging with semiconductor quantum dots. Nature Biotechnology 2004, 22, (8), 969-976.

5. Wu, X. Y.; Liu, H. J.; Liu, J. Q.; Haley, K. N.; Treadway, J. A.; Larson, J. P.; Ge, N. F.; Peale, F.; Bruchez, M. P., Immunofluorescent labeling of cancer marker Her2 and other cellular targets with semiconductor quantum dots. Nature Biotechnology 2003, 21, (1), 41-46.
